# Recent advances in coarse-grained simulations of nucleic acid condensation

**DOI:** 10.1042/BCJ20260251

**Published:** 2026-10-15

**Authors:** Jessica Fong Ng, Shanlong Li, Jianhan Chen

**Affiliations:** 1 Department of Chemistry, University of Massachusetts, Amherst, MA 01003, U.S.A .; 2 Molecular and Cellular Biology Program, University of Massachusetts, Amherst, MA 01003, U.S.A.

**Keywords:** coarse-graining, dynamic structure, ion coordination, molecular simulation, phase transition

## Abstract

Biomolecular condensates, formed through phase separation of multivalent biomolecules, play essential roles in cellular processes such as stress response, RNA processing, transcriptional control, and genome organization. Nucleic acids are increasingly recognized as important drivers and regulators of condensate formation and dynamics. This review examines recent advances in coarse-grained molecular simulations of dynamic nucleic acids and their phase transitions. We survey established computational frameworks validated against experimental data and discuss emerging applications that address increasingly complex systems. Existing studies reveal that base-mediated interactions emerge as dominant drivers in homotypic nucleic acid condensation and that simulation resolution fundamentally determines which physical phenomena are accessible and observable. We underscore that different modeling approaches at various resolutions provide complementary insights into nucleic acid condensation mechanisms and the driving forces. We emphasize the opportunities and challenges in extending these frameworks to capture the complexity of nucleic acid-mediated phase separation in biological contexts, while identifying important gaps in current modeling capabilities. Overall, molecular simulations at multiple scales have proven to be powerful tools for elucidating the mechanistic principles underlying nucleic acid condensates and their cellular functions.

## Introduction

Biomolecular condensates, also known as membraneless organelles, are an important mechanism of subcellular organization and have a wide range of physiological functions [1–4]. Their broad relevance to both fundamental biology and human disease has made them a subject of intense interdisciplinary research interest in recent years [5–7]. They are formed through spontaneous phase transitions of dynamic multivalent molecules such as proteins, DNAs, and RNAs, driven by a wide range of weak intermolecular interactions, including electrostatics, hydrophobic interactions, hydrogen bonding, aromatic stacking, and π–cation interactions [2,8–11]. Biomolecular condensates display rich material properties ranging from liquid-like to gel and solid-like [12,13], which have important consequences in function [14–16] and have also been implicated in the pathology of neurodegenerative and other human diseases [17,18].

While protein-driven condensation has received much attention, nucleic acids are key components of many biomolecular condensates, including ribonucleoprotein granules, stress granules, and chromatin-associated compartments. Phase separation provides a fundamental mechanism to organize and regulate nucleic acids across genomic organization [19], support transcription regulation [20] and stress response [21,22]. This mechanism has also been implicated in some neurodegeneration, where disease manifests only beyond a critical number of trinucleotide repeat expansions within specific genes [23–25]. Beyond biological systems, nucleic acid phase separation has emerged as a promising avenue for synthetic biology and drug delivery applications [26,27].

Nucleic acids exhibit phase behaviors that are physically distinct from proteins [13]. First, RNAs typically undergo lower critical solution temperature (LCST) phase transitions (Figure 1A), entropically driven by dehydration and counterion release [28]. Reentrant transitions at high temperature have also been reported, revealing both upper critical solution temperature (UCST)- and LCST-type behaviors [28,29]. Furthermore, these transitions can be reversible or irreversible, depending on RNA sequence and concentrations, and Mg^2+^ level, with reversibility likely determined by propensity to percolate and form intra-condensate networks (Figure 1A) [28]. Second, homotypic nucleic acid phase separation generally requires crowding agents, divalent cations, or polycationic species, underscoring the central role of ion-mediated interactions in charge neutralization and inter-chain bridging (Figure 1B) [13,30]. Third, RNA trinucleotide repeats phase separate more readily than their DNA counterparts, a disparity hypothesized to stem from the additional 2′-OH on the ribose ring, which facilitates inner-sphere coordination with Mg^2+^ and promotes water release [30,31]. In heterotypic systems, nucleic acids introduce additional regulatory complexity: RNA can act as both a promoter and inhibitor of condensation through reentrant phase transitions, where excess RNA overcharges condensate droplets and induces dissolution [13,29]. Together, these observations emphasize that nucleic acid condensation is governed by a delicate balance of electrostatic interactions, base-mediated contacts (stacking and pairing), backbone chemistry, and ion coordination (Figure 1A–C), modulated by sequence, composition, and solution conditions.

**Figure 1:**
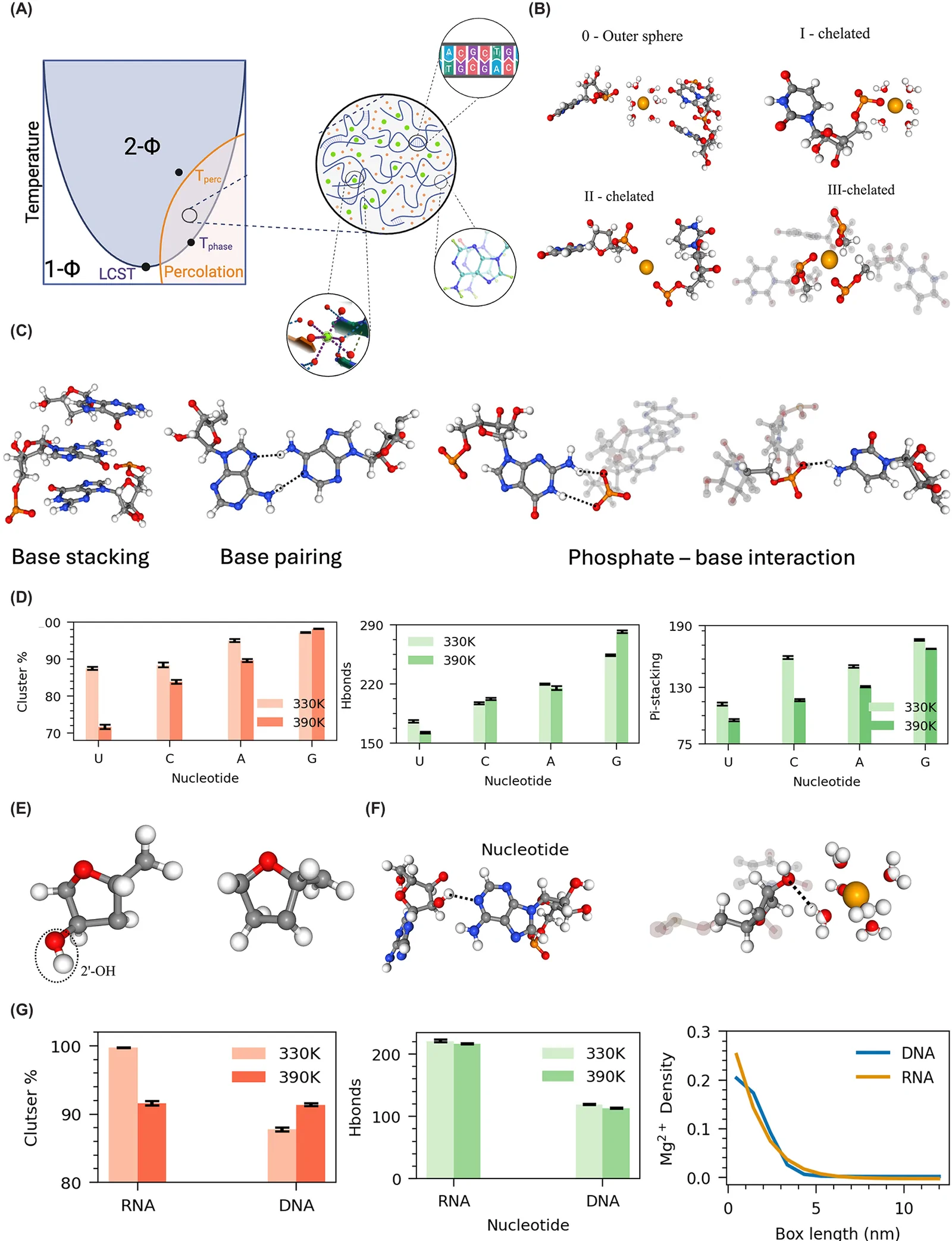
Interactions in nucleic acid-mediated condensation (**A**) A schematic phase diagram of LCST-type phase transitions of nucleic acids, with key interactions present in the phase-separated (2-φ) and percolation regimes summarized on the right. (**B**) Mg^2+^-dependent coordination of phosphate backbone interactions. (**C**) Nucleobase-mediated interactions. (**D**) Comparison of average RNA percentages in the cluster (left), hydrogen bonds (middle), and π-stacking (right) for G_4_, A_4_, C_4_, and U_4_ at 330K and 390K from atomistic simulations. (**E**) Chemical structure of sugar ring for RNA (left) and DNA (right). (**F**) 2′-OH-mediated interactions. (**G**) Comparison of average rA_4_ or dA_4_ percentages in the cluster (left), hydrogen bonds (middle), and Mg^2+^ density in /nm^3^ (right) from atomistic simulations at 330K and 390K. Panels B-G are reproduced from ref. [30] with permission. Copyright 2025 National Academy of Sciences.

Elucidating these molecular driving forces experimentally remains challenging. Techniques such as turbidity assays, fluorescence microscopy, and SAXS can characterize macroscopic phase boundaries and global structural parameters, but they cannot directly resolve the interplay of competing interactions at the molecular level. For example, how Mg^2+^ coordination modes shift with temperature, how base stacking competes with electrostatic bridging within condensates, or how individual chain conformations reorganize upon entering the dense phase. Molecular dynamics simulations offer a uniquely powerful complement by providing direct access to these microscopic details [32] and have provided valuable insights into the underlying mechanisms and driving forces [33–35]. However, phase separation involves length and timescales that are largely inaccessible to all-atom simulations. Coarse-grained (CG) models have therefore become essential to bridge this gap in a computationally efficient manner. Models spanning different resolutions, ranging from minimal models to one-bead-per-nucleotide and multi-bead-per-nucleotide representations, have been developed in conjunction with experiments and theory to elucidate the mechanisms and regulation of biomolecular condensates [13,33,34,36].

In this review, we examine recent developments in molecular modeling of flexible RNAs and DNAs and their phase transitions, with particular emphasis on efficient CG models. We begin by discussing insights from atomistic simulations that reveal the key driving forces and establish physical constraints for CG model development. We then survey CG models organized by resolution, from minimal and patchy-particle representations to one-bead, two-bead, and intermediate multi-bead models, discussing their parameterization strategies, validated capabilities, and application to both homotypic nucleic acid condensation and heterotypic protein–nucleic acid systems. We highlight emerging applications to structurally complex systems, including G-quadruplexes and nucleic acid nanostars, and conclude by identifying key gaps and future directions. Models developed primarily for nucleic acid structure prediction [37], chromatin organization [36,38,39], and earlier CG frameworks for nucleic acid simulations [36,39–42] have been reviewed elsewhere and are beyond the scope of this work.

## Atomistic insights into nucleic acid phase transitions

Atomistic simulations using empirical physics-based force fields arguably provide the most detailed description of the molecular interactions involved in biomolecular and particularly nucleic acid condensation. Although direct simulation of macroscopic phase separation remains mostly beyond computational reach at fully atomic resolution, targeted studies of small oligonucleotide systems have proven valuable for dissecting the relative contributions of specific driving forces, including ion coordination, base-mediated contacts, hydration, and backbone chemistry, under conditions relevant to condensation. These simulations highlight interaction features that existing CG models may inadequately capture, providing guidance for CG model refinement.

Distinct force fields have been developed for RNA (e.g. AMBER χOL3 [43], CHARMM36 [44]) and DNA (e.g. AMBER BSC1 [45], AMBER OL24 [46], and CHARMM36 [44]); both face similar challenges in accurately capturing the balance of electrostatic, stacking, and solvent-mediated interactions relevant to condensation. For nucleic acid phase separation specifically, the force field must simultaneously describe electrostatic interactions between negatively charged phosphate groups and cations, base-mediated interactions, including base pairing and stacking, and the thermodynamics of hydration and ion coordination (Figure 1A–C). Achieving this balance has proven challenging, as shown in ongoing reparameterization efforts [35] and systematic benchmarking studies [47–51]. For comprehensive evaluation of nucleic acid force fields and associated water models, we refer readers to recent reviews [35,52] and benchmarking studies against experimental data [47–50]. Despite these challenges, carefully selected force field combinations have yielded important mechanistic insights into nucleic acid condensation, as highlighted below.

The polyphosphate (poly(P)) system offers a minimal model to isolate the role of backbone-ion interactions in condensation, independent of base-mediated contributions. Banerjee and coworkers demonstrated experimentally that poly(P) readily undergoes LCST-type phase separation in the presence of Mg^2+^ ions [28], where atomistic simulations using microMg [53] and TIP3P water model [54] were also performed to reveal the underlying mechanism: elevated temperature promotes entropically favorable desolvation of both phosphate groups and Mg^2+^ ions, which in turn enhances inter-chain cross-linking and drives chain compaction and network formation characteristic of phase separation. This establishes a clear physical picture in which thermally activated dehydration of ion-phosphate contacts provides the entropic driving force for LCST behavior. Interestingly, poly(P) can undergo phase separation with monovalent ions experimentally [55]. That is, divalent cross-linking is not strictly required, and charge neutralization alone is sufficient. However, neither DNA nor RNA undergoes phase separation with monovalent ions alone; they require additional divalent ions [28,56,57] or polycation species [58,59] to condense. This distinction indicates that for nucleic acids containing bases, backbone charge neutralization is necessary but not sufficient—additional interactions mediated by the base and/or the sugar are required.

Building on this foundation, Ramachandran and Potoyan applied a carefully selected combination of AMBER RNA force field parameters (parmBSC0χOL3) [46,60,61], microMg [53], and the OPC water model [62] to dissect the molecular determinants of phase separation in small RNA and DNA oligonucleotides. Their simulations revealed that three factors collectively tune condensate stability: Mg^2+^ coordination, nucleobase chemistry, and 2′-hydroxyl group (2′-OH) (Figure 1D–G) [30]. Beyond heat-driven Mg^2+^ binding and water-released mechanism identified in polyphosphate, the study uncovered a thermal stability hierarchy among RNA homopolymers: poly(G) > poly(A) > poly(C) > poly(U) that predicts their relative propensity to phase separate through coupled ion and hydration effects (Figure 1D). This hierarchy reflects differences in base-mediated interactions: guanine and adenine engage in more stacking and hydrogen bonding networks that provide additional cohesive forces beyond electrostatic bridging. The 2′-OH group on the RNA ribose ring further enhances condensation propensity by enabling hydrogen bonding interactions with Mg^2+^ and intramolecular base contacts (Figure 1F,G). This leads to a higher propensity for RNA phase separation compared with DNA, consistent with experimental observation [31].

Together, these atomistic studies reveal that nucleic acid condensation arises from a delicate interplay of ion coordination, base-mediated contacts, backbone hydration, and sugar chemistry. The relative importance of these contributions varies with sequence, temperature, and ionic conditions, giving rise to the rich phase behaviors observed experimentally. These findings provide a useful physical context for assessing and developing CG models, which we survey in the following section.

## CG models for nucleic acid phase separation

While atomistic simulations reveal detailed dynamics, they cannot access the length and time scales required to directly simulate phase separation. CG models bridge this gap by reducing the degrees of freedom while retaining essential physical features. Many CG nucleic acid models have been developed, spanning resolutions from minimal representations to intermediate multi-bead frameworks (Table 1). These models employ diverse parameterization strategies, drawing on atomistic simulations, experimental structural data on dynamic nucleic acids, thermodynamic measurements, and statistical potentials, and have been applied to both homotypic nucleic acid condensation and heterotypic protein–nucleic acid systems. However, there is a lack of a systematic benchmark to assess these models against different experimental observables for single-stranded RNA (ssRNA), single-stranded DNA (ssDNA), phase separation properties, and computing resources.

**Table 1:** Overview of coarse-grained nucleic acid models in phase separation, their coarse-graining schemes, and parameterization strategies

Model	Coarse-graining scheme	Parameterization strategy
Tangade et al., 2025 [63]	1 bead per 1 nucleotide for ssDNA; 1 bead per 1 base-pair for dsDNA	Experimental protein–DNA interaction strength
Karmakar et al., 2024 [64]	1 bead per 2 nucleotides	Experimental salt-dependent Experimental ssRNA *L*_p_
Joseph et al., 2021 [65]	RNA-binding protein/RNA as hard sphere with attractive sites, RNA as self-avoiding polymers	Experimental [RNA] dependent FUS *C*_sat_
HPS [66] (RNA)	1 bead per nucleotide	atomic partial charges from OPLS all-atom RNA force field [67]
SIS [68]	1 bead per nucleotide	(CAG)_2_ duplex structure, statistical base-pairing and dihedral potentials
Mpipi (Recharge) [69,70]	1 bead per nucleotide	Atomistic PMFs for nonbonded interactions
COCOMO [71]	1 bead per nucleotide	Experimental salt dependent ssRNA structural properties
NEAT-DNA [72]	1 bead per nucleotide	Atomistic and MD simulation on duplex and nucleosome; SAXS
HPS-Urry (DNA) [73]	1 backbone bead + 1 base bead	atomic scale partial charges from CHARMM27 all-atom force field [74]; Experimental duplex melting temperatures, dsDNA structural properties and protein–DNA interaction strength
CALVADOS [75,76]	1 backbone bead (at P) + 1 base bead (at N connected to sugar)	Experimental salt dependence of polyA_30_ and polyU_30_ SAXS
iConRNA [77]	3 backbone beads (at P, C1′ C4′ of sugar ring) + 3 pyrimidine base beads and 4 purine base beads	Statistical potential of relative stacking and base-pairing strengths [78]; Experimental ssRNA structural properties, and T_m_ of small RNAs
Martini-based [79]	3 backbone beads + 7 guanine/cytosine beads	Length-dependent phase separation propensity

In this section, we will first focus on CG models designed for homotypic nucleic acid phase separations, where we highlight two models, namely SIS and iConRNA, that together build a coherent picture of how base-mediated interactions emerge as the dominant driving force of condensation.

### Single-site and triple-site interaction models (SIS/TIS)

SIS is a model pioneered by Thirumalai’s group [68], where each nucleotide is represented as a single interaction center. The model has been employed to investigate how systems like riboswitches respond to mechanical force [80,81]. SIS incorporates bonded interactions (bond and angle terms), excluded volume, and base pairing interactions, but no explicit electrostatic forces. Instead, it is calibrated for conditions where high concentrations of monovalent and divalent ions effectively screen phosphate charges [82,83], thereby enabling phase separation. The only adjustable parameter is the energy scale for base pairing interaction, and it was calibrated to reproduce the crystal structures of short (CAG)_2_ duplex [68]. This minimalist parameterization—a single energy scale targeting a single structural observable—makes the model’s subsequent successes in capturing emergent phase behavior particularly noteworthy.

Multichain simulations using the calibrated SIS successfully recapitulated the length and concentration dependence of CAG repeat phase separation observed experimentally [25]. The results suggest that, within the condensed phase, intramolecular base pairings are substantially reduced, and dynamic intermolecular base pairings become dominant and allow the formation of a dynamic, soft network composed of stretched and unfolded ssRNA. This condensate-induced structural reorganization, where the dense phase environment reshapes the balance between intra- and intermolecular contacts, has been observed in other contexts experimentally [84]. The model further revealed heterogeneous condensate architecture and identified reptation-like chain dynamics, associating RNA secondary structure reorganization with condensate dynamics. These results established a critical insight: base pairing, even when limited to canonical Watson–Crick contacts, is sufficient to drive phase separation and determine condensate internal organization when electrostatic screening is high.

However, because the SIS model captured only base pairing without electrostatics and ion coordination, several important questions remain unaddressed. What is the relative contribution of electrostatic binding versus base-mediated contacts? Is base pairing alone sufficient, or do other base interactions—particularly stacking—play an equally important role? How does Mg^2+^ contribute beyond simple charge screening? These questions require a model that explicitly incorporates both electrostatic and base-mediated interactions at sufficient resolution to distinguish between them.

Extensions of the SIS model have incorporated electrostatic interactions through the Debye–Hückel (DH) approximation to describe implicit monovalent ions [85,86] with explicit divalent ions [87]. Further developments include the sequence-dependent three-interaction site (TIS) model [88] and its derivative TIS-CAT [89], which employs reference interaction site model (RISM)-based potential mean forces of divalent cations-phosphate interactions featuring distinct inner-sphere and outer-sphere coordination minima. These models incorporate explicit representation of monovalent salts and/or divalent cations and have been employed to study diverse RNA and DNA folding [90]. While TIS-CAT has not yet been applied to phase separation directly, its explicit treatment of Mg^2+^ coordination makes it a promising framework for future studies of ion-dependent condensation.

### Intermediate resolution model for condensates of RNAs (iConRNA)

To explicitly capture the balance of diverse interactions of RNA backbone, side chain base, and ions, Li and Chen developed the iConRNA model with intermediate representation of each nucleotide and explicit Mg^2+^ ions [77]. Each nucleotide is represented by 6–7 beads: one bead for the phosphate, two beads for the sugar ring, and three to four beads for the pyrimidine or purine base, respectively (Figure 2A). This resolution enables the model to incorporate explicit electrostatic, base stacking, and base pairing interactions, such that one could carefully tune the balance of their relative contributions to structure dynamics and condensation. Mg^2+^ ions are represented as spheres corresponding to the fully hydrated state and interact nonspecifically with the base and sugar groups and electrostatically with the phosphate beads. Since Mg^2+^ binding with RNA depends strongly on monovalent salt concentration, sequence, structure, and temperature [91], the model employs an empirical scaling factor λ for the phosphate-Mg^2+^ electrostatic interaction strength, calibrated using excess magnesium per phosphate (Δ*n*_mg_) [91–94] determined through atomistic simulations, theory, machine learning approaches, or experiments for a given RNA sequence.

**Figure 2:**
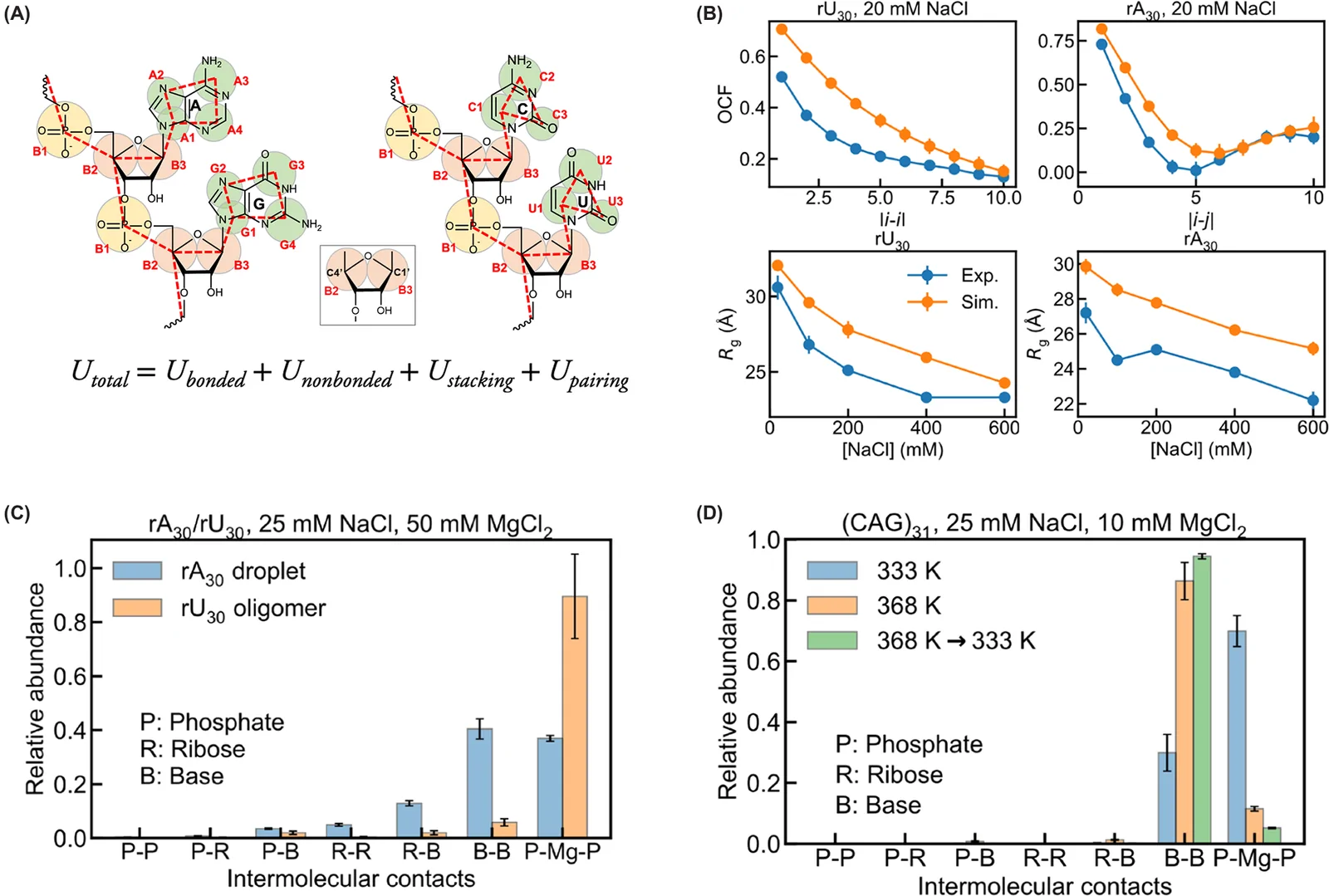
Condensate enhances intermolecular interactions (**A**) CG mapping and energy function of iConRNA [77]. (**B**) OCF and Rg of poly(rA)30 and poly(rU)30. Relative abundance of different intermolecular interactions found in (**C**) rA30 droplets and rU oligomers and (**D**) (CAG) 31 condensates simulated under different temperatures. 368 K→333 K indicates condensates obtained from annealing those originally formed at 368 K to 333 K. Panels (A–D) are reproduced from ref. [77] with permission. Copyright 2026 National Academy of Sciences.

iConRNA has been carefully balanced to reproduce local and long-range structures of flexible ssRNAs, including backbone orientation correlation function (OCF), radius of gyration (*R*_g_), and end-to-end distance (*R*_e_) under different salt and Mg^2+^ concentrations (Figure 2B), as well as folding and melting temperature of a set of small RNAs. This multi-target parameterization ensures the balance between interactions is reasonable and transferable for phase separation studies. The model was shown to capture the experimentally observed distinction between rA_30_ and rU_30_, where rA_30_ can readily undergo phase separation with 50 mM MgCl_2_ at elevated temperature, while poly(rU) remains homogenous under similar conditions [95]. Crucially, the analysis revealed that, although rU_30_ exhibits stronger Mg^2+^ bridging interactions than rA_30_ (Figure 2C), this electrostatic-mediated stabilization alone is not sufficient to drive condensation. The critical difference lies in the base-mediated interactions: interchain Van der Waals interactions and intermolecular base stacking present in rA_30_ provided the additional driving forces necessary for phase separation (Figure 2C). Examination of rA_30_ structure within the dense phase revealed reduced structural complexity compared with dilute conditions, reflecting weakening of intramolecular interactions and enhanced intermolecular interactions, consistent with the SIS observations for CAG repeats [68].

A notable strength of iConRNA is the ability to accurately recapitulate the nontrivial dependence of CAG repeat phase separation on sequence length, ionic strength, temperature, Mg^2+^, and RNA concentrations [25,28]. This is a major achievement that has not been demonstrated by other CG or atomistic models to date. The model similarly captured the phase diagrams of purine- to uracil-substituted trinucleotide repeats: CAG and CUG repeats undergo phase separation while the CUU repeat does not under comparable conditions. Across all these systems, base–base interactions remain the dominant driving force even at elevated temperatures, outweighing increases in phosphate-Mg^2+^ interaction strength. This dominance is also observed through the annealing protocol, where the condensate was cooled to 333K from 368K; base-base interactions retain substantial strength, suggesting they serve as the driving force of condensate gelation and arrest (Figure 2D).

Nonetheless, important questions remain. Can the iConRNA framework be extended to DNA and RNA–DNA hybrid systems? Does the current resolution adequately distinguish their differential phase separation propensities? The iConRNA model currently represents Mg^2+^ only in the fully hydrated state without distinguishing inner-sphere from outer-sphere coordination modalities identified in atomistic simulations (Figure 1B) and only includes canonical base pairing and neighboring base-stacking. Incorporation of these additional interactions will likely be necessary for further improving the model’s phase separation predictive capabilities. Another important direction is the integration of iConRNA with an appropriate protein model, such as the hybrid resolution (HyRes) model [96–99], towards modeling heterotypic phase transitions.

## Emerging application of CG models to phase separation of complex nucleic acids

The CG models discussed above have primarily been applied to simple nucleic acid sequences such as homopolymers and trinucleotide repeats, which behave largely as flexible single-stranded polymers with only transient local structures. However, nucleic acids can adopt complex geometries that influence phase separation in biologically and technologically important ways [100,101]. While applications of CG modeling to phase transitions of RNAs with complex structures remain limited, emerging studies have begun to address this gap. We highlight two examples, namely, G-quadruplexes and engineered nucleic acid nanostars. However, CG models have yet to be applied to functional RNAs and long noncoding RNAs, representing an exciting frontier for future research.

### G-quadruplex

Nucleic acids rich in guanine can fold into four-stranded G-quadruplex (G4) structures stabilized by Hoogsten hydrogen base-pairing and coordinated by central ions [102]. The phase transition of G4C2 RNA repeats is directly implicated in neurodegenerative pathologies, including familial amyotrophic lateral sclerosis and frontotemporal dementia, where disease manifests only beyond a critical repeat length [25]. Elucidating how G4 formations coupled to condensation is therefore of significant biomedical interest.

Souza’s group adapted the Martini 3 force field [103] to address this challenge. To capture the hydrogen bonding and π–π stacking driving G4 architecture, they expanded the standard nucleobase resolution from 3–4 beads to 7 beads in guanine and cytosine [79] with explicit ions (including Mg^2+^) from Martini 3 [103]. The model can capture the spontaneous quadruplex folding without relying on an elastic network. In multi-chain simulations, increasing G4C2 repeat length from 3 to 8 caused a decrease in average cluster count alongside increased chain compactness, mirroring experimental observations that viable condensate formation *in vivo* required thresholds beyond five G4C2 repeats [25]. The simulations revealed significant enrichment of Mg^2+^ in the dense phase relative to Na^+^ and Cl^−^, consistent with multi-valent Mg^2+^-phosphate interactions. The present study demonstrated the potential of CG modeling for connecting non-canonical RNA structure formation to phase behavior, even though further validation, including temperature and salt dependence, and condensate material properties remain to be explored.

### Nucleic acid nanostars

Synthetic DNA and RNA nanostars, multi-armed structures interacting via sequence-dependent kissing loops, have emerged as programmable scaffolds for engineering artificial condensates [104–106]. These structures can undergo phase separation *in vitro* without magnesium and *in vivo*. They can be designed with specific valencies, arm lengths, and binding motifs to create orthogonal non-mixing condensates with tunable material properties. Their programmability makes them attractive systems for both synthetic biology and as model systems to test physical theories of valency-dependent phase transitions.

Many models at different resolutions have been used to study DNA nanostar dynamics [107–109] and phase separation [110–113]. Here, we focus on a recent study that compared the flexibility of DNA and RNA nanostars using two-bead models oxRNA [114,115] and oxDNA [116–120], which represent each nucleotide as a rigid body with anisotropic interaction sites. It investigated how valency, salt, and temperature differentially control nanostar geometry versus flexibility [121]. These models consist of one backbone bead and three interaction sites on the base bead for base repulsion, base stacking, and hydrogen bond/cross-stacking. They have been extensively parameterized to reproduce thermodynamics and mechanistic properties of RNA [114,115] and DNA [116–120]. The study revealed that increasing the number of arms does not alter individual arm flexibility and that DNA nanostars exhibited larger deviations in inter-arm angles compared with their RNA counterparts. This observation supported the ability of these models to capture backbone-dependent differences in RNA and DNA dynamics. Extending these frameworks to multichain phase separation simulations represents an exciting opportunity to connect programmable nanostar design with condensate formation and properties.

## CG models for protein–nucleic acid heterotypic phase transitions

Beyond homotypic nucleic acid systems, many biomolecular condensates involve protein–nucleic acid co-condensation, where electrostatic complementarity between positively charged protein regions and negatively charged nucleic acid backbone is a primary driving force. These systems display rich phase behaviors such as reentrant phase transition [122,123], charge inversion [124], and multiphasic architectures [123]. Many CG nucleic acid models have been developed to complement existing protein models in studying these phenomena. These models range from minimalist models representing entire proteins and nucleic acids as single particles or patchy particles to models with multiple beads per nucleotide (Table 1). They have been calibrated using different strategies to capture various physical aspects of the protein/nucleic acid phase behaviors. Below we discuss representative models within increasing resolution, with particular attention to the nucleic acid component.

### Minimal models and patchy particles

At the coarsest level, minimal continuum models, including patchy-particle models and lattice-based approaches (notably “stickers-and-spacers” representations), have been used to study intrinsically disordered protein phase behaviors, revealing concentration-dependent features of multicomponent protein systems [9,125–130]. Similar approaches have also been used to study RNA–protein [65] and DNA–histone interactions [63,131] (Table 1). These studies revealed that complementary electrostatic interactions and relative protein–protein and protein–nucleic acid binding strengths can tune condensate formation and compartmentalization. However, these models lack residue-level details and cannot address sequence-specific effects.

### Nucleotide-based one-bead models

One-bead per nucleotide models offer a practical compromise, representing each nucleotide with a single interaction site while retaining sequence identity (Figure 3A). Prominent examples include HPS [66], Mpipi/Mpipi-Recharge [69,70], and COCOMO [71]. These one-bead models emphasize the electrostatic contributions: each bead carries some effective negative charge (−1 in HPS and COCOMO, −0.75 in Mpipi), with electrostatic interactions typically described using the DH potential. The latest Mpipi-Recharge model introduced asymmetric charge–charge interactions using a pair-specific Yukawa potential derived from reference potentials of mean force (PMFs) [70], allowing one to differentiate between the electrostatic behaviors of similarly charged residues (e.g. arginine versus lysine) in condensation and providing a better description of the highly charged interfaces of multi-domain proteins and ssRNAs. Cation–π interactions between basic protein residues and aromatic nucleic acid bases are often effectively captured through Lennard-Jones-style or Wang–Frenkel nonbonded interactions, such as in COCOMO and Mpipi, respectively.

**Figure 3:**
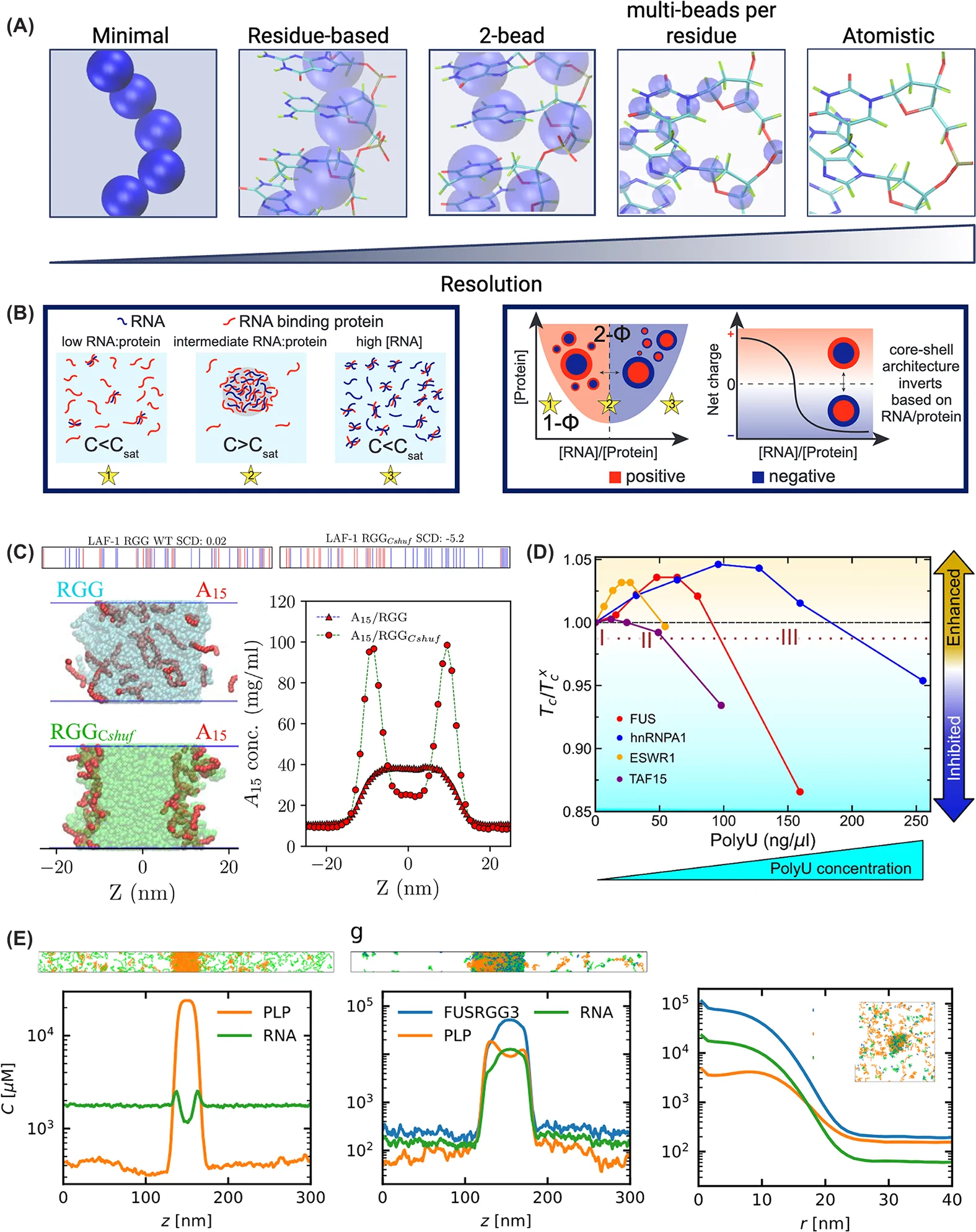
Protein–nucleic acid interactions and phase transitions (**A**) CG models of nucleic acids with increasing resolutions, ranging from minimal to atomistic resolution. (**B**) RNA-dependent reentrant behavior (left). The increase of [RNA] enhances phase separation initially but becomes inhibitory when RNA concentration exceeds the neutralization point. The phase diagram (right) depicts the two-phase (2-Φ) and one-phase (1-Φ) regions as a function of RNA/protein ratio. Net charge of the condensate determines the core-shell architecture, which inverts depending on the RNA/protein ratio. (**C**) RNA localization depends on protein sequence charge patterning. Two sequences with different charge patterning are shown at the top (LAF-1 RGG, SCD = −0.02; and RGGC_shuf_, SCD = −5.2), where lower SCD indicates increased charge segregation. Slab simulations (bottom left) showed A_15_ RNA (red) partitioned into the condensed phase for RGG (cyan) and RGGC_shuf_ (green). Density profiles of A_15_ along the z-axis (bottom right) confirmed enhanced RNA recruitment with increased charge patterning. (**D**) Poly(rU)-dependent reentrant phase behavior. (**E**) Preferential binding of RNA to RBP compared with PLP sequences. Panel (B) is reproduced from ref. [13] with permission. Copyright 2024, The Authors, licensed via Copyright Clearance Center. Panel (C) is reproduced from ref. [66] with permission under the terms of the Open Access CC BY-NC license. Copyright 2020. Panel (D) is reproduced from ref. [70] with permission under the terms of Creative Commons CC BY 4.0. Copyright 2025. Panel (E) is reproduced from [75] with permission. Copyright 2025, The Authors, licensed via Copyright Clearance Center.

Different parameterization strategies have been designed for optimization of various one-bead models. For example, HPS utilizes atomic-scale partial charges from the OPLS all-atom RNA force field to calculate the hydropathy value that determines interaction strengths, resulting in repulsive nucleotide–nucleotide homotypic interactions, while attraction toward positively charged amino acids is significant [66]. Mpipi model derived nonbonded parameters from atomic PMFs for all nucleobase-nucleobase and nucleobase-amino acid pairs [69,70]. COCOMO incorporated a top-down approach, parameterizing directly against experimental data, including *R*_g_, OCF, *R*_e_, and persistence length (*L*_p_) of ssRNA homopolymers [132,133]. COCOMO achieved good agreement across many properties, except for the OCF of homopolymer adenine, which likely requires explicit base stacking absent from the one-bead model.

Both HPS and Mpipi-Recharge have been successfully applied to study the re-entrant behavior with RRP sequences and RNA [122,124] (Figure 3B). Simulations using HPS showed that with patches of acidic and basic sequences present in the LAF-1 RGG sequence, the RNA partition is preferentially at the surface, where it mainly interacts with the positive patches [66] (Figure 3C). In addition, increasing RNA concentration first reduces C_sat_ of condensate and then increases C_sat_ beyond a certain point, suggesting both promoter and disruptor roles of RNA in condensate formation. Mpipi-Recharge reproduced similar trends across several arginine-rich polypeptide (RRP) sequences with poly(rU) (Figure 3D), demonstrating that asymmetric treatment of electrostatic interactions improves the recapitulation of reentrant phase behavior with gradual increase of poly(rU) concentration [70]. COCOMO similarly produced condensate clusters in several RNA–protein systems [71]. Nonetheless, the one-bead resolution imposes fundamental limitations; it severely restricts the ability to capture transient local structures (e.g. base stacking), the balance of backbone and side chain interactions in protein–nucleic phase transitions, or conformational rearrangement of nucleic acid within condensates.

### Backbone-base two-bead models

These models represent each nucleotide with one backbone bead carrying −1 charge and one neutral base bead (Figure 3A), allowing explicit separation of backbone electrostatics from base interactions. A representative two-bead model is HPS-Urry DNA [73], which was developed to model duplex DNA interacting with proteins in chromatin contexts [73,134]. It includes stacking of consecutive bases and canonical base pairing, with interaction parameters derived from CHARMM27 [74] partial charges and PMF calculations [135], adjusted to reproduce the melting profile of a 14-bp DNA duplex. The model reproduced dsDNA structural properties, including base rise, helical width, base pairs per turn, and major and minor grooves. Protein–DNA interactions are validated through the dissociation constant (*K*_d_) of Histone H1 C-terminus with a 20 bp dsDNA. HPS-Urry protein and DNA models together can thus provide a framework for modeling phase transitions in chromatin studies.

CALVADOS-RNA [75] is another representative two-bead model, but designed for flexible RNA polymers to be used alongside the CALVADOS single-bead protein model [136]. The interactions are sequence-independent, with “stickiness” parameters (λ) adjusted to reproduce poly-rU_30_ and poly-(rA)_30_ *R*_g_ under different salt concentrations. Interestingly, the model also captured the compactness of poly-(dT)_30_ and poly-(dA)_30_ from SAXS at low salt concentration (225 mM), though it produces overly compact structures at higher salt conditions (525 mM) [137]. This suggests that CALVADOS-RNA is potentially a general nucleic acid model appropriate for both ssRNAs and ssDNAs under physiological salt conditions (e.g. ∼150 mM). The model can capture the re-entrant behavior observed experimentally, in which the lowest concentration of protein for phase separation is near the neutralization point and returns to one phase when RNA concentration is high [123]. In the FUS^RGG3^, PLP, and polyU system, the model can also recapitulate the preferential partition of RNA with FUS^RGG3^ as shown in experiment [123].

### Models with three or more beads per nucleotide

These models represent each nucleotide with one phosphate bead, one sugar bead, and one to multiple base beads [77,79,138,139] (Figure 3A). While many models have been applied to study nucleic acid folding with explicit divalent ions [90,138,139] and nucleic acid phase separation [68,77,79], most have not been used for heterotypic protein–nucleic acid phase separation. We note that the Martini force field has compatible protein and nucleic acid parameters, providing a ready-made framework for multicomponent condensation studies and a significant opportunity for the field [103,140]. A challenge, though, is to capture transient protein and nucleic structures that likely play important roles in modulating phase transitions and condensate properties.

## Concluding discussion and outlook

In this review, we have surveyed molecular models developed for modeling nucleic acid phase separation, spanning from all-atom simulations to CG representations at various resolutions. Atomistic simulations have provided critical mechanistic insights into the driving forces of condensation—phosphate-ion interactions, nucleobase-mediated interactions, and solvent reorganization—but remain limited to small systems. CG models aim to bridge this gap and have demonstrated expanding capabilities for capturing experimentally observed phase behaviors, including LCST-type transitions, sequence- and length-dependent condensation, reentrant dissolution in heterotypic systems, and condensate spatial organization.

A key theme emerging across model resolution is the trade-off between computational efficiency and physical fidelity. Minimal, one-bead and two-bead models can efficiently explore large sequence and composition spaces, and capture phenomena governed primarily by electrostatic interaction, such as reentrant phase behavior, RNA-mediated condensate regulation, and preferential partitioning in protein–RNA condensates, but cannot access the divalent ion- and base-mediated driving forces that atomistic and intermediate-resolution models have identified as major driving forces in homotypic systems. Intermediate-resolution models such as iConRNA can include explicit electrostatic, stacking, and pairing interactions and achieve superior agreement across a range of experimental observables, including sequence, length, temperature, ion, and concentration dependence on phase separation. Together, this growing toolkit of models at complementary resolutions provides the community with flexible approaches suited to different questions of biological, biomedical, and bioengineering interests.

The physical picture established by atomistic simulation provides a useful lens for assessing the current state of CG modeling. Several features identified as important at the atomistic level remain challenging to capture in most CG frameworks. First, Mg^2+^ coordination involves distinct inner-sphere and outer-sphere modalities with different thermodynamic signatures. Most CG models treat Mg^2+^ either implicitly through DH screening or as a single hydrated sphere, without distinguishing these coordination modes. Second, the 2′-OH group on the RNA ribose ring contributes measurably to condensation propensity by enabling additional hydrogen bonding with ions and bases. Third, the LCST characteristic of nucleic acid phase transitions arises from temperature-dependent dehydration, an inherently solvent-mediated effect. Developing strategies to encode this into implicit-solvent CG frameworks would expand their applicability to temperature-dependent studies.

Looking forward, several directions appear particularly promising. First, a systematic characterization of protein–nucleic acid interactions at the molecular level remains a critical gap. Interactions such as cation–π contacts between basic protein residues and nucleobases, electrostatic complementarity between arginine/lysine-rich regions and phosphate backbones, hydrogen bonding at the protein–RNA interfaces, and counter-ion release collectively govern nucleic acid recruitment and condensate spatial organization. Experimental thermodynamics data for individual protein–nucleic acid interactions remain sparse. As these data become available, they will enable more rigorous parameterization beyond combination rules and PMF calculations, improving the transferability across different protein–nucleic acid systems.

Second, extending current models to distinguish RNA from DNA phase behavior is underexplored. The differential condensation propensities of RNA and DNA oligomers have been experimentally observed, and atomistic simulations have hypothesized 2′-OH as a key contributor. Incorporating this distinction into CG frameworks, either through explicit representation of sugar chemistry [141] or through differential effective parameterization at lower resolution, would enable the study of RNA–DNA hybrid systems and their biological roles.

Third, improved treatment of divalent ion interactions would benefit models across different resolutions. The RISM-based approach employed in TIS-CAT, which captures both inner-sphere and outer-sphere coordination, offers one path forward. Alternatively, machine learning approaches trained on atomistic ion coordination data could provide transferable effective potentials.

Fourth, the connection between molecular interactions and condensate material properties is also an important area. The observation from iConRNA that base-base interactions increased after annealing suggests that these contacts can potentially drive gelation and irreversible droplet formation, indicating that models capturing these interactions can potentially predict not only phase boundaries, but also liquid-to-solid transitions implicated in diseases. Systematic studies connecting CG model parameters to rheological observables would open an exciting bridge between simulation and experiment.

Finally, the growing availability of experimental data on nucleic acid condensate structure, composition, and dynamics creates opportunities for more rigorous model parametrization and validation. Quantitative measurements, including SAXS-derived structural properties, single-molecule FRET, NMR characterization of ion environments, and rheological data, can serve as multi-target benchmarks for model development, moving the field toward increasingly predictive and transferable frameworks. The convergence of improved experimental characterization, advancing computational resources, and emerging machine learning approaches [72,142,143] provides exciting opportunities for the field to deliver more comprehensive models to study biomolecular phase separation in increasingly cell-like environments.

## Open Access

Open access for this article was enabled by the participation of the University of Massachusetts - Amherst in an all-inclusive Read & Publish agreement with Portland Press and the Biochemical Society.

## Abbreviations

CG: Coarse-grained
DH: Debye–Hückel
LCST: Lower critical solution temperature
OCF: Orientation correlation function
PMFs: Potentials of mean force
RISM: Reference interaction site model
SIS: Single-site interaction model
ssRNA: Single-stranded RNA
UCST: Upper critical solution temperature

## References

[1] Brangwynne C.P., Eckmann C.R., Courson D.S., Rybarska A., Hoege C., Gharakhani J. et al. (2009) Germline P granules are liquid droplets that localize by controlled dissolution/condensation. Science 324, 1729–1732 19460965 10.1126/science.1172046

[2] Li P., Banjade S., Cheng H.C., Kim S., Chen B., Guo L. et al. (2012) Phase transitions in the assembly of multi-valent signaling proteins. Nature 483, 336–340 22398450 10.1038/nature10879 PMC3343696

[3] Banani S.F., Lee H.O., Hyman A.A. and Rosen M.K. (2017) Biomolecular condensates: organizers of cellular biochemistry. Nat. Rev. Mol. Cell Biol. 18, 285–298 28225081 10.1038/nrm.2017.7 PMC7434221

[4] Mangiarotti A. and Dimova R. (2026) Phase separation across membranes and condensates in cell organization and function. Nat. Rev. Mol. Cell Biol. 27, 564–580 41957206 10.1038/s41580-026-00961-5

[5] Alberti S., Gladfelter A. and Mittag T. (2019) Considerations and challenges in studying liquid–liquid phase separation and biomolecular condensates. Cell 176, 419–434 30682370 10.1016/j.cell.2018.12.035 PMC6445271

[6] Klein I.A., Boija A., Afeyan L.K., Hawken S.W., Fan M., Dall'Agnese A. et al. (2020) Partitioning of cancer therapeutics in nuclear condensates. Science 368, 1386–1392 32554597 10.1126/science.aaz4427 PMC7735713

[7] Alberti S., Arosio P., Best R.B., Boeynaems S., Cai D., Collepardo-Guevara R. et al. (2025) Current practices in the study of biomolecular condensates: a community comment. Nat. Commun. 16, 7730 40830340 10.1038/s41467-025-62055-8 PMC12365244

[8] Alberti S. and Dormann D. (2019) Liquid–liquid phase separation in disease. Annu. Rev. Genet. 53, 171–194 31430179 10.1146/annurev-genet-112618-043527

[9] Martin E.W., Holehouse A.S., Peran I., Farag M., Incicco J.J., Bremer A. et al. (2020) Valence and patterning of aromatic residues determine the phase behavior of prion-like domains. Science 367, 694–699 32029630 10.1126/science.aaw8653 PMC7297187

[10] Pappu R.V., Cohen S.R., Dar F., Farag M. and Kar M. (2023) Phase transitions of associative biomacromolecules. Chem. Rev. 123, 8945–8987 36881934 10.1021/acs.chemrev.2c00814 PMC11513790

[11] Holehouse A.S. and Kragelund B.B. (2024) The molecular basis for cellular function of intrinsically disordered protein regions. Nat. Rev. Mol. Cell Biol. 25, 187–211 37957331 10.1038/s41580-023-00673-0 PMC11459374

[12] Mittag T. and Pappu R.V. (2022) A conceptual framework for understanding phase separation and addressing open questions and challenges. Mol. Cell. 82, 2201–2214 35675815 10.1016/j.molcel.2022.05.018 PMC9233049

[13] Wadsworth G., Srinivasan S., Lai L., Datta M., Gopalan V. and Banerjee P.R. (2024) RNA-driven phase transitions in biomolecular condensates. Mol. Cell. 84, 3692–3705 39366355 10.1016/j.molcel.2024.09.005 PMC11604179

[14] Lee M., Moon H.C., Jeong H., Kim D.W., Park H.Y. and Shin Y. (2024) Optogenetic control of mRNA condensation reveals an intimate link between condensate material properties and functions. Nat. Commun. 15, 3216 38622120 10.1038/s41467-024-47442-x PMC11018775

[15] Das T., Zaidi F.K., Farag M., Ruff K.M., Mahendran T.S., Singh A. et al. (2025) Tunable metastability of condensates reconciles their dual roles in amyloid fibril formation. Mol. Cell. 85, 2230–2245.e7 40441157 10.1016/j.molcel.2025.05.011 PMC12831641

[16] Jia B., Shen Z., Zhu S., Huang J., Liao Z., Zhao S. et al. (2025) Shank3 oligomerization governs material properties of the postsynaptic density condensate and synaptic plasticity. Cell 188, 6473–6491.e21 40848728 10.1016/j.cell.2025.07.047

[17] Alberti S. and Hyman A.A. (2021) Biomolecular condensates at the nexus of cellular stress, protein aggregation disease and ageing. Nat. Rev. Mol. Cell Biol. 22, 196–213 33510441 10.1038/s41580-020-00326-6

[18] Lyon A.S., Peeples W.B. and Rosen M.K. (2021) A framework for understanding the functions of biomolecular condensates across scales. Nat. Rev. Mol. Cell Biol. 22, 215–235 33169001 10.1038/s41580-020-00303-z PMC8574987

[19] Feric M. and Misteli T. (2021) Phase separation in genome organization across evolution. Trends Cell Biol. 31, 671–685 33771451 10.1016/j.tcb.2021.03.001 PMC8286288

[20] Stortz M., Presman D.M. and Levi V. (2024) Transcriptional condensates: a blessing or a curse for gene regulation? Commun. Biol. 7, 187 38365945 10.1038/s42003-024-05892-5 PMC10873363

[21] Pessina F., Giavazzi F., Yin Y., Gioia U., Vitelli V., Galbiati A. et al. (2019) Functional transcription promoters at DNA double-strand breaks mediate RNA-driven phase separation of damage-response factors. Nat. Cell Biol. 21, 1286–1299 31570834 10.1038/s41556-019-0392-4 PMC6859070

[22] Franzmann T.M., Jahnel M., Pozniakovsky A., Mahamid J., Holehouse A.S., Nüske E. et al. (2018) Phase separation of a yeast prion protein promotes cellular fitness. Science 359, eaao5654 29301985 10.1126/science.aao5654

[23] La Spada A.R. and Taylor J.P. (2010) Repeat expansion disease: progress and puzzles in disease pathogenesis. Nat. Rev. Genet. 11, 247–258 20177426 10.1038/nrg2748 PMC4704680

[24] Krzyzosiak W.J., Sobczak K., Wojciechowska M., Fiszer A., Mykowska A. and Kozlowski P. (2012) Triplet repeat RNA structure and its role as pathogenic agent and therapeutic target. Nucleic. Acids. Res. 40, 11–26 21908410 10.1093/nar/gkr729 PMC3245940

[25] Jain A. and Vale R.D. (2017) RNA phase transitions in repeat expansion disorders. Nature 546, 243–247 28562589 10.1038/nature22386 PMC5555642

[26] Fabrini G., Farag N., Nuccio S.P., Li S., Stewart J.M., Tang A.A. et al. (2024) Co-transcriptional production of programmable RNA condensates and synthetic organelles. Nat. Nanotechnol. 19, 1665–1673 39080489 10.1038/s41565-024-01726-x PMC11567899

[27] Chen W., Fritzen J. and Walther A. (2026) Phase separation of nucleic acids: mechanisms, properties, and applications. Angew. Chem. Int. Ed. 65, e23943 10.1002/anie.202523943 PMC12970503 41636358

[28] Wadsworth G.M., Zahurancik W.J., Zeng X., Pullara P., Lai L.B., Sidharthan V. et al. (2023) RNAs undergo phase transitions with lower critical solution temperatures. Nat. Chem. 15, 1693–1704 37932412 10.1038/s41557-023-01353-4 PMC10872781

[29] Pullara P., Alshareedah I. and Banerjee P.R. (2022) Temperature-dependent reentrant phase transition of RNA-polycation mixtures. Soft Matter 18, 1342–1349 34984429 10.1039/D1SM01557E PMC8854377

[30] Ramachandran V. and Potoyan D.A. (2025) Molecular drivers of RNA phase separation. Proc. Natl. Acad. Sci. U.S.A. 122, e2511348122 41187075 10.1073/pnas.2511348122 PMC12625925

[31] Wadsworth G.M., Aierken D., Thurston G.M., Joseph J.A. and Banerjee P.R. (2026) The role of the 2′-OH group in phase separation and percolation transitions of RNA. Nat. Commun. 17 9296 42669726 10.1038/s41467-026-75961-2 PMC13527071

[32] Brangwynne C.P., Tompa P. and Pappu R.V. (2015) Polymer physics of intracellular phase transitions. Nat. Phys. 11, 899–904 10.1038/nphys3532

[33] Maristany M.J., Emelianova A., Chew P.Y., Aguirre A., Collepardo-Guevara R. and Joseph J.A. (2025) Modeling biomolecular condensates across scales: atomistic, coarse-grained, and data-driven approaches. Adv. Phys. X 10, 2592547 41868013 10.1080/23746149.2025.2592547 PMC13004485

[34] Chew P.Y. and Collepardo-Guevara R. (2025) Probing molecular and biophysical mechanisms of RNA and protein phase transitions with simulations and theory. Curr. Opin. Struct. Biol. 93, 103120 40664121 10.1016/j.sbi.2025.103120

[35] Languin-Cattoën O. and Bussi G. (2026) RNA dynamics and interactions revealed through atomistic simulations. Annu. Rev. Phys. Chem. 77, 489–512 41729696 10.1146/annurev-physchem-082624-013453

[36] Korolev N., Nordenskiöld L. and Lyubartsev A.P. (2016) Multiscale coarse-grained modelling of chromatin components: DNA and the nucleosome. Adv. Colloid Interface Sci. 232, 36–48, 26956528 10.1016/j.cis.2016.02.002

[37] Li J. and Chen S.J. (2021) RNA 3D structure prediction using coarse-grained models. Front Mol. Biosci. 8, 720937 34277713 10.3389/fmolb.2021.720937 PMC8283274

[38] Zhou R. and Gao Y.Q. (2020) Polymer models for the mechanisms of chromatin 3D folding: review and perspective. Phys. Chem. Chem. Phys. 22, 20189–20201 32966415 10.1039/D0CP01877E

[39] Korolev N., Sun T., Lyubartsev A.P. and Nordenskiöld L. (2025) Coarse-grained modeling of electrostatic interactions in chromatin. WIREs Comput. Mol. Sci. 15, e70059 10.1002/wcms.70059

[40] Dawson W.K., Maciejczyk M., Jankowska E.J. and Bujnicki J.M. (2016) Coarse-grained modeling of RNA 3D structure. Methods 103, 138–156, 27125734 10.1016/j.ymeth.2016.04.026

[41] Sun T., Minhas V., Korolev N., Mirzoev A., Lyubartsev A.P. and Nordenskiöld L. (2021) Bottom-up coarse-grained modeling of DNA. Front Mol. Biosci. 8, 645527 33816559 10.3389/fmolb.2021.645527 PMC8010198

[42] Li W. and Chen S.J. (2026) Building RNA coarse-grained force fields: design principles and training strategies. Biophys. J. 125, 4898–4912 41877485 10.1016/j.bpj.2026.03.041 PMC13110076

[43] Mlýnský V., Kührová P., Stadlbauer P., Krepl M., Otyepka M., Banáš P. et al. (2023) Simple adjustment of intranucleotide base-phosphate interaction in the OL3 AMBER force field improves RNA simulations. J. Chem. Theory Comput. 19, 8423–8433 37944118 10.1021/acs.jctc.3c00990 PMC10687871

[44] Denning E.J., Priyakumar U.D., Nilsson L. and MacKerell A.D. (2011) Impact of 2′-hydroxyl sampling on the conformational properties of RNA: update of the CHARMM all-atom additive force field for RNA. J. Comput. Chem. 32, 1929–1943 21469161 10.1002/jcc.21777 PMC3082605

[45] Ivani I., Dans P.D., Noy A., Pérez A., Faustino I., Hospital A. et al. (2016) Parmbsc1: a refined force field for DNA simulations. Nat. Methods 13, 55–58 26569599 10.1038/nmeth.3658 PMC4700514

[46] Pérez A., Marchán I., Svozil D., Sponer J., Cheatham T.E., Laughton C.A. et al. (2007) Refinement of the AMBER force field for nucleic acids: improving the description of α/γ conformers. Biophys. J. 92, 3817–3829 17351000 10.1529/biophysj.106.097782 PMC1868997

[47] Sarthak K., Winogradoff D., Ge Y., Myong S. and Aksimentiev A. (2023) Benchmarking molecular dynamics force fields for all-atom simulations of biological condensates. J. Chem. Theory Comput. 19, 3721–3740 37134270 10.1021/acs.jctc.3c00148 PMC11169342

[48] Winkler L. and Cheatham T.E. (2023) Benchmarking the Drude polarizable force field using the r(GACC) tetranucleotide. J. Chem. Inf. Model. 63, 2505–2511 36996447 10.1021/acs.jcim.3c00250 PMC10131218

[49] Mlýnský V., Kührová P., Pykal M., Krepl M., Stadlbauer P., Otyepka M. et al. (2025) Can we ever develop an ideal RNA force field? Lessons learned from simulations of the UUCG RNA tetraloop and other systems J. Chem. Theory Comput. 21, 4183–4202 39813107 10.1021/acs.jctc.4c01357 PMC12020377

[50] Lemmens T., Mlýnský V., Šponer J., Pykal M., Banáš P., Otyepka M. et al. (2025) The kink-turn 7 motif: an additional test for RNA force field performance. J. Chem. Theory Comput. 21, 12796–12809 41358869 10.1021/acs.jctc.5c00776 PMC12746442

[51] Love O., Galindo-Murillo R., Zgarbová M., Šponer J., Jurečka P. and Cheatham T.E. (2023) Assessing the current state of AMBER force field modifications for DNA—2023 edition. J. Chem. Theory Comput. 19, 4299–4307 37340948 10.1021/acs.jctc.3c00233 PMC10339676

[52] Liebl K. and Zacharias M. (2023) The development of nucleic acids force fields: from an unchallenged past to a competitive future. Biophys. J. 122, 2841–2851 36540025 10.1016/j.bpj.2022.12.022 PMC10398263

[53] Grotz K.K., Cruz-León S. and Schwierz N. (2021) Optimized magnesium force field parameters for biomolecular simulations with accurate solvation, ion-binding, and water-exchange properties. J. Chem. Theory Comput. 17, 2530–2540 33720710 10.1021/acs.jctc.0c01281 PMC8047801

[54] Jorgensen W.L., Chandrasekhar J., Madura J.D., Impey R.W. and Klein M.L. (1983) Comparison of simple potential functions for simulating liquid water. J. Chem. Phys. 79, 926–935 10.1063/1.445869

[55] Furuki T., Togo A., Usuda H., Nobeyama T., Hirano A. and Shiraki K. (2024) Monovalent ion effect on liquid–liquid phase separation of aqueous polyphosphate–salt mixtures. J. Phys. Chem. B. 128, 11435–11440 39508447 10.1021/acs.jpcb.4c03457

[56] Merindol R., Loescher S., Samanta A. and Walther A. (2018) Pathway-controlled formation of mesostructured all-DNA colloids and superstructures. Nat. Nanotechnol. 13, 730–738 29941888 10.1038/s41565-018-0168-1 PMC6082344

[57] Liu W., Samanta A., Deng J., Akintayo C.O. and Walther A. (2022) Mechanistic insights into the phase separation behavior and pathway‐directed information exchange in all‐DNA droplets. Angew. Chem. Int. Ed. 61, e202208951 10.1002/anie.202208951 PMC9828218 36112754

[58] Shakya A. and King J.T. (2018) DNA local-flexibility-dependent assembly of phase-separated liquid droplets. Biophys. J. 115, 1840–1847 30342746 10.1016/j.bpj.2018.09.022 PMC6303412

[59] Vieregg J.R., Lueckheide M., Marciel A.B., Leon L., Bologna A.J., Rivera J.R. et al. (2018) Oligonucleotide–peptide complexes: phase control by hybridization. J. Am. Chem. Soc. 140, 1632–1638 29314832 10.1021/jacs.7b03567

[60] Banáš P., Hollas D., Zgarbová M., Jurečka P., Orozco M., Cheatham T.E. III et al. (2010) Performance of molecular mechanics force fields for RNA simulations: stability of UUCG and GNRA hairpins. J. Chem. Theory Comput. 6, 3836–3849 35283696 10.1021/ct100481h PMC8916691

[61] Zgarbová M., Otyepka M., Šponer J., Mládek A., Banáš P., Cheatham T.E. III et al. (2011) Refinement of the Cornell et al. nucleic acids force field based on reference quantum chemical calculations of glycosidic torsion profiles. J. Chem. Theory Comput. 7, 2886–2902 21921995 10.1021/ct200162x PMC3171997

[62] Grotz K.K. and Schwierz N. (2022) Magnesium force fields for OPC water with accurate solvation, ion-binding, and water-exchange properties: successful transfer from SPC/E. J. Chem. Phys. 156, 114501 35317575 10.1063/5.0087292

[63] Tangade A.S., Mondal A., Wang J., Kim Y.C. and Mittal J. (2025) Multiphasic organization and differential dynamics of proteins within protein–DNA biomolecular condensates. J. Phys. Chem. B. 129, 9588–9599 40954121 10.1021/acs.jpcb.5c03987 PMC12478860

[64] Karmakar S.D. and Speck T. (2024) Dependencies between effective parameters in coarse-grained models for phase separation of DNA-based fluids. J. Chem. Phys. 161, 234907 39692499 10.1063/5.0232651

[65] Joseph J.A., Espinosa J.R., Sanchez-Burgos I., Garaizar A., Frenkel D. and Collepardo-Guevara R. (2021) Thermodynamics and kinetics of phase separation of protein–RNA mixtures by a minimal model. Biophys. J. 120, 1219–1230 33571491 10.1016/j.bpj.2021.01.031 PMC8059209

[66] Regy R.M., Dignon G.L., Zheng W., Kim Y.C. and Mittal J. (2020) Sequence dependent phase separation of protein-polynucleotide mixtures elucidated using molecular simulations. Nucleic. Acids. Res. 48, 12593–12603 33264400 10.1093/nar/gkaa1099 PMC7736803

[67] Robertson M.J., Qian Y., Robinson M.C., Tirado-Rives J. and Jorgensen W.L. (2019) Development and testing of the OPLS-AA/M force field for RNA. J. Chem. Theory Comput. 15, 2734–2742 30807148 10.1021/acs.jctc.9b00054 PMC6585454

[68] Nguyen H.T., Hori N. and Thirumalai D. (2022) Condensates in RNA repeat sequences are heterogeneously organized and exhibit reptation dynamics. Nat. Chem. 14, 775–785 35501484 10.1038/s41557-022-00934-z

[69] Joseph J.A., Reinhardt A., Aguirre A., Chew P.Y., Russell K.O., Espinosa J.R. et al. (2021) Physics-driven coarse-grained model for biomolecular phase separation with near-quantitative accuracy. Nat. Comput. Sci. 1, 732–743 35795820 10.1038/s43588-021-00155-3 PMC7612994

[70] Tejedor A.R., Aguirre Gonzalez A., Maristany M.J., Chew P.Y., Russell K., Ramirez J. et al. (2025) Chemically informed coarse-graining of electrostatic forces in charge-rich biomolecular condensates. ACS Cent Sci. 11, 302–321 40028356 10.1021/acscentsci.4c01617 PMC11869137

[71] Valdes-Garcia G., Heo L., Lapidus L.J. and Feig M. (2023) Modeling concentration-dependent phase separation processes involving peptides and RNA via residue-based coarse-graining. J. Chem. Theory Comput. 19, 669–678 10.1021/acs.jctc.2c00856 PMC10323037 36607820

[72] Riveros I. and Zhang B. (2026) NEAT-DNA: a chemically accurate, sequence-dependent coarse-grained model for large-scale DNA simulations. J. Chem. Theory Comput. 22, 3709–3719 41875330 10.1021/acs.jctc.5c01966 PMC13047904

[73] Kapoor U., Kim Y.C. and Mittal J. (2024) Coarse-grained models to study protein–DNA interactions and liquid–liquid phase separation. J. Chem. Theory Comput. 20, 1717–1731 37988476 10.1021/acs.jctc.3c00525 PMC10911113

[74] MacKerell A.D. Jr and Banavali N.K. (2000) All-atom empirical force field for nucleic acids: II. Application to molecular dynamics simulations of DNA and RNA in solution. J. Comput. Chem. 21, 105–120 10.1002/(SICI)1096-987X(20000130)21:2<105::AID-JCC3>3.0.CO;2-P

[75] Yasuda I., von Bülow S., Tesei G., Yamamoto E., Yasuoka K. and Lindorff-Larsen K. (2025) Coarse-grained model of disordered RNA for simulations of biomolecular condensates. J. Chem. Theory Comput. 21, 2766–2779 40009520 10.1021/acs.jctc.4c01646

[76] Ramachandran V. and Potoyan D.A. (2026) A molecular grammar for programmable multiphase protein–RNA vesicles. JACS Au 6, 3310–3322 42358691 10.1021/jacsau.6c00322 PMC13292008

[77] Li S. and Chen J. (2025) Driving forces of RNA condensation revealed through coarse-grained modeling with explicit Mg^2+^. Proc. Natl. Acad. Sci. U.S.A. 122, e2504583122 41129218 10.1073/pnas.2504583122 PMC12582263

[78] Li J. and Chen S.J. (2023) RNAJP: enhanced RNA 3D structure predictions with non-canonical interactions and global topology sampling. Nucleic. Acids. Res. 51, 3341–3356 36864729 10.1093/nar/gkad122 PMC10123122

[79] Zhang Q., Valério M., Grünewald L., Borges-Araújo L., Grünewald F., Wang S. et al. (2026) Coarse-grained simulations reveal salt- and length-dependent condensation of G4C2 RNA repeats. J. Phys. Chem. Lett. 17, 6445–6454 42223083 10.1021/acs.jpclett.6c00916

[80] Lin J.C. and Thirumalai D. (2008) Relative stability of helices determines the folding landscape of adenine riboswitch aptamers. J. Am. Chem. Soc. 130, 14080–14081 18828635 10.1021/ja8063638

[81] Lin J.C. and Thirumalai D. (2013) Kinetics of allosteric transitions in S-adenosylmethionine riboswitch are accurately predicted from the folding landscape. J. Am. Chem. Soc. 135, 16641–16650 24087850 10.1021/ja408595e PMC3861500

[82] Manning G.S. (1978) The molecular theory of polyelectrolyte solutions with applications to the electrostatic properties of polynucleotides. Q. Rev. Biophys. 11, 179–246 353876 10.1017/S0033583500002031

[83] Bloomfield V.A. (1997) DNA condensation by multivalent cations. Biopolymers 44, 269–282 9591479 10.1002/(SICI)1097-0282(1997)44:3<269::AID-BIP6>3.0.CO;2-T

[84] Rangadurai A.K., Ruetz L., Ahmed R., Lo K., Tollinger M., Forman-Kay J.D. et al. (2024) Phase separation modulates the thermodynamics and kinetics of RNA hybridization. J. Am. Chem. Soc. 146, 19686–19689 38991204 10.1021/jacs.4c06530

[85] Maity H., Nguyen H.T., Hori N. and Thirumalai D. (2023) Odd-even disparity in the population of slipped hairpins in RNA repeat sequences with implications for phase separation. Proc. Natl. Acad. Sci. U.S.A. 120, e2301409120 37276412 10.1073/pnas.2301409120 PMC10268303

[86] Maity H., Nguyen H.T., Hori N. and Thirumalai D. (2024) Salt-dependent self-association of trinucleotide repeat RNA sequences. J. Phys. Chem. Lett. 15, 3820–3827 38557079 10.1021/acs.jpclett.3c03553

[87] Maity H., Zhang H., Thirumalai D. and Nguyen H.T. (2025) RNA structural complexity dictates its ion atmosphere. J. Phys. Chem. Lett. 16, 8393–8402 40773731 10.1021/acs.jpclett.5c01656

[88] Denesyuk N.A. and Thirumalai D. (2015) How do metal ions direct ribozyme folding? Nat Chem. 7, 793–801 26391078 10.1038/nchem.2330

[89] Nguyen H.T., Hori N. and Thirumalai D. (2019) Theory and simulations for RNA folding in mixtures of monovalent and divalent cations. Proc. Natl. Acad. Sci. U.S.A. 116, 21022–21030 31570624 10.1073/pnas.1911632116 PMC6800359

[90] Thirumalai D., Hori N. and Nguyen H.T. (2025) Minimal models for RNA simulations. Curr. Opin. Struct. Biol. 93, 103107 40616978 10.1016/j.sbi.2025.103107

[91] Misra V.K. and Draper D.E. (1999) The interpretation of Mg^2+^ binding isotherms for nucleic acids using Poisson–Boltzmann theory1. J. Mol. Biol. 294, 1135–1147 10600372 10.1006/jmbi.1999.3334

[92] Bai Y., Greenfeld M., Travers K.J., Chu V.B., Lipfert J., Doniach S. et al. (2007) Quantitative and comprehensive decomposition of the ion atmosphere around nucleic acids. J. Am. Chem. Soc. 129, 14981–14988 17990882 10.1021/ja075020g PMC3167487

[93] Jacobson D.R. and Saleh O.A. (2017) Counting the ions surrounding nucleic acids. Nucleic. Acids. Res. 45, 1596–1605 28034959 10.1093/nar/gkw1305 PMC5389524

[94] Xi K., Wang F.H., Xiong G., Zhang Z.L. and Tan Z.J. (2018) Competitive binding of Mg^2+^ and Na^+^ Ions to nucleic acids: from helices to tertiary structures. Biophys. J. 114, 1776–1790 29694858 10.1016/j.bpj.2018.03.001 PMC5936997

[95] Tom J.K.A., Onuchic P.L. and Deniz A.A. (2022) Short polyA RNA homopolymers undergo Mg^2+^-mediated kinetically arrested condensation. J. Phys. Chem. B. 126, 9715–9725 36378781 10.1021/acs.jpcb.2c05935 PMC9706566

[96] Liu X. and Chen J. (2017) HyRes: a coarse-grained model for multi-scale enhanced sampling of disordered protein conformations. Phys. Chem. Chem. Phys. 19, 32421–32432 29186229 10.1039/C7CP06736D PMC5729119

[97] Zhang Y., Liu X. and Chen J. (2022) Toward accurate coarse-grained simulations of disordered proteins and their dynamic interactions. J. Chem. Inf. Model. 62, 4523–4536 36083825 10.1021/acs.jcim.2c00974 PMC9910785

[98] Zhang Y., Li S., Gong X. and Chen J. (2024) Toward accurate simulation of coupling between protein secondary structure and phase separation. J. Am. Chem. Soc. 146, 342–357 38112495 10.1021/jacs.3c09195 PMC10842759

[99] Li S., Barethiya S. and Chen J. (2026) HyRes: accurate physics-based simulation of dynamic protein structures and interactions in complex environments at scale. bioRxiv 2026.06.23.734133, Available from: https://www.biorxiv.org/content/10.64898/2026.06.23.734133v142395354

[100] Langdon E.M., Qiu Y., Ghanbari Niaki A., McLaughlin G.A., Weidmann C.A., Gerbich T.M. et al. (2018) mRNA structure determines specificity of a polyQ-driven phase separation. Science 360, 922–927 29650703 10.1126/science.aar7432 PMC6192030

[101] Roden C. and Gladfelter A.S. (2021) RNA contributions to the form and function of biomolecular condensates. Nat. Rev. Mol. Cell Biol. 22, 183–195 32632317 10.1038/s41580-020-0264-6 PMC7785677

[102] Varshney D., Spiegel J., Zyner K., Tannahill D. and Balasubramanian S. (2020) The regulation and functions of DNA and RNA G-quadruplexes. Nat. Rev. Mol. Cell Biol. 21, 459–474 32313204 10.1038/s41580-020-0236-x PMC7115845

[103] Souza P.C.T., Alessandri R., Barnoud J., Thallmair S., Faustino I., Grünewald F. et al. (2021) Martini 3: a general purpose force field for coarse-grained molecular dynamics. Nat. Methods 18, 382–388 33782607 10.1038/s41592-021-01098-3 PMC12554258

[104] Li S., Kim Y., Wang K., Payson E.J., Tang A.A., Villalba Nieto M. et al. (2026) Programmable artificial RNA condensates in mammalian cells. Nat. Nanotechnol. 21, 821–830 42056202 10.1038/s41565-026-02164-7 PMC13293865

[105] Aposhian J., Deopa S.P.S., Horowitz S. and Yesselman J.D. (2026) Self-assembling RNA nanostructures are highly sensitive to environmental conditions. J. Mol. Biol. 438, 169601 41422840 10.1016/j.jmb.2025.169601 PMC13288444

[106] Skipper K. and Wickham S.F.J. (2026) DNA nanostars that self-assemble into core-shell condensate microdroplets. Nanoscale Horiz 11, 1320–1331 41738383 10.1039/D5NH00788G

[107] Lee T., Do S., Lee J.G., Kim D.N. and Shin Y. (2021) The flexibility-based modulation of DNA nanostar phase separation. Nanoscale 13, 17638–17647 34664044 10.1039/D1NR03495B

[108] Gutiérrez Fosado Y.A. (2023) Nanostars planarity modulates the rheology of DNA hydrogels. Soft Matter 19, 4820–4828 37226672 10.1039/D2SM00221C PMC10321217

[109] Gadzekpo A. and Hilbert L. (2026) Bayesian optimisation and graph-based rheology enable sequence-dependent modelling of DNA materials. bioRxiv 2026.05.27.728076, Available from: https://www.biorxiv.org/content/10.64898/2026.05.27.728076v1

[110] Rovigatti L., Smallenburg F., Romano F. and Sciortino F. (2014) Gels of DNA nanostars never crystallize. ACS Nano 8, 3567–3574 24646041 10.1021/nn501138w

[111] Locatelli E., Handle P.H., Likos C.N., Sciortino F. and Rovigatti L. (2017) Condensation and demixing in solutions of DNA nanostars and their mixtures. ACS Nano 11, 2094–2102 28157331 10.1021/acsnano.6b08287 PMC5333195

[112] Agarwal S., Osmanovic D., Klocke M.A. and Franco E. (2022) The growth rate of DNA condensate droplets increases with the size of participating subunits. ACS Nano 16, 11842–11851 35867936 10.1021/acsnano.2c00084

[113] Liu S. and Košmrlj A. (2025) Predicting the interfacial energy and morphology of DNA condensates. arXiv Available from: http://arxiv.org/abs/2512.16150

[114] Šulc P., Romano F., Ouldridge T.E., Doye J.P.K. and Louis A.A. (2014) A nucleotide-level coarse-grained model of RNA. J. Chem. Phys. 140, 235102 24952569 10.1063/1.4881424

[115] Matek C., Šulc P., Randisi F., Doye J.P.K. and Louis A.A. (2015) Coarse-grained modelling of supercoiled RNA. J. Chem. Phys. 143, 243122 26723607 10.1063/1.4933066

[116] Snodin B.E.K., Schreck J.S., Romano F., Louis A.A. and Doye J.P.K. (2019) Coarse-grained modelling of the structural properties of DNA origami. Nucleic. Acids. Res. 47, 1585–1597 30605514 10.1093/nar/gky1304 PMC6379721

[117] Ouldridge T.E., Louis A.A. and Doye J.P.K. (2010) DNA nanotweezers studied with a coarse-grained model of DNA. Phys. Rev. Lett. 104, 178101 20482144 10.1103/PhysRevLett.104.178101

[118] Ouldridge T.E., Louis A.A. and Doye J.P.K. (2011) Structural, mechanical, and thermodynamic properties of a coarse-grained DNA model. J. Chem. Phys. 134, 085101 21361556 10.1063/1.3552946

[119] Šulc P., Romano F., Ouldridge T.E., Rovigatti L., Doye J.P.K. and Louis A.A. (2012) Sequence-dependent thermodynamics of a coarse-grained DNA model. J. Chem. Phys. 137, 135101 23039613 10.1063/1.4754132

[120] Snodin B.E.K., Randisi F., Mosayebi M., Šulc P., Schreck J.S., Romano F. et al. (2015) Introducing improved structural properties and salt dependence into a coarse-grained model of DNA. J. Chem. Phys. 142, 234901 26093573 10.1063/1.4921957

[121] Kadam P.R., Lim J., Dizani M. and Stewart J.M. (2026) Coarse-grained modeling and simulation of multistranded RNA nanostars. Chem. Bio. Eng. 3, 37–48 41602950 10.1021/cbe.5c00093 PMC12833626

[122] Banerjee P.R., Milin A.N., Moosa M.M., Onuchic P.L. and Deniz A.A. (2017) Reentrant phase transition drives dynamic substructure formation in ribonucleoprotein droplets. Angew. Chem. Int. Ed. 56, 11354–11359 10.1002/anie.201703191 PMC5647147 28556382

[123] Kaur T., Raju M., Alshareedah I., Davis R.B., Potoyan D.A. and Banerjee P.R. (2021) Sequence-encoded and composition-dependent protein–RNA interactions control multiphasic condensate morphologies. Nat. Commun. 12, 872 33558506 10.1038/s41467-021-21089-4 PMC7870978

[124] Alshareedah I., Kaur T., Ngo J., Seppala H., Kounatse L.A.D., Wang W. et al. (2019) Interplay between short-range attraction and long-range repulsion controls reentrant liquid condensation of ribonucleoprotein–RNA complexes. J. Am. Chem. Soc. 141, 14593–14602 31437398 10.1021/jacs.9b03689 PMC7069731

[125] Harmon T.S., Holehouse A.S. and Pappu R.V. (2018) Differential solvation of intrinsically disordered linkers drives the formation of spatially organized droplets in ternary systems of linear multivalent proteins. New J. Phys. 20, 045002 10.1088/1367-2630/aab8d9

[126] Harmon T.S., Holehouse A.S., Rosen M.K. and Pappu R.V. (2017) Intrinsically disordered linkers determine the interplay between phase separation and gelation in multivalent proteins. eLife 6, e30294 29091028 10.7554/eLife.30294 PMC5703641

[127] Nguemaha V. and Zhou H.X. (2018) Liquid–liquid phase separation of patchy particles illuminates diverse effects of regulatory components on protein droplet formation. Sci. Rep. 8, 6728 29712961 10.1038/s41598-018-25132-1 PMC5928213

[128] Ghosh A., Mazarakos K. and Zhou H.X. (2019) Three archetypical classes of macromolecular regulators of protein liquid–liquid phase separation. Proc. Natl. Acad. Sci. U.S.A. 116, 19474–19483 31506351 10.1073/pnas.1907849116 PMC6765290

[129] Choi J.M., Dar F. and Pappu R.V. (2019) LASSI: a lattice model for simulating phase transitions of multivalent proteins. PLoS Comput. Biol. 15, e1007028 31634364 10.1371/journal.pcbi.1007028 PMC6822780

[130] Espinosa J.R., Joseph J.A., Sanchez-Burgos I., Garaizar A., Frenkel D. and Collepardo-Guevara R. (2020) Liquid network connectivity regulates the stability and composition of biomolecular condensates with many components. Proc. Natl. Acad. Sci. U.S.A. 117, 13238–13247 32482873 10.1073/pnas.1917569117 PMC7306995

[131] Kang H., Yoo J., Sohn B.K., Lee S.W., Lee H.S., Ma W. et al. (2018) Sequence-dependent DNA condensation as a driving force of DNA phase separation. Nucleic. Acids. Res. 46, 9401–9413 30032232 10.1093/nar/gky639 PMC6182145

[132] Chen H., Meisburger S.P., Pabit S.A., Sutton J.L., Webb W.W. and Pollack L. (2012) Ionic strength-dependent persistence lengths of single-stranded RNA and DNA. Proc. Natl. Acad. Sci. U.S.A. 109, 799–804 22203973 10.1073/pnas.1119057109 PMC3271905

[133] Plumridge A., Andresen K. and Pollack L. (2020) Visualizing disordered single-stranded RNA: connecting sequence, structure, and electrostatics. J. Am. Chem. Soc. 142, 109–119 31804813 10.1021/jacs.9b04461 PMC7238861

[134] Regy R.M., Thompson J., Kim Y.C. and Mittal J. (2021) Improved coarse‐grained model for studying sequence dependent phase separation of disordered proteins. Protein Sci. Publ. Protein Soc. 30, 1371–1379 10.1002/pro.4094 PMC8197430 33934416

[135] Shankar A., Jagota A. and Mittal J. (2012) DNA base dimers are stabilized by hydrogen-bonding interactions including non-Watson–Crick pairing near graphite surfaces. J. Phys. Chem. B. 116, 12088–12094 22967176 10.1021/jp304260t

[136] Norgaard A.B., Ferkinghoff-Borg J. and Lindorff-Larsen K. (2008) Experimental parameterization of an energy function for the simulation of unfolded proteins. Biophys. J. 94, 182–192 17827232 10.1529/biophysj.107.108241 PMC2134871

[137] Sim A.Y.L., Lipfert J., Herschlag D. and Doniach S. (2012) Salt dependence of the radius of gyration and flexibility of single-stranded DNA in solution probed by small-angle x-ray scattering. Phys. Rev. E. 86, 021901 10.1103/PhysRevE.86.021901 23005779

[138] Freeman G.S., Hinckley D.M. and de Pablo J.J. (2011) A coarse-grain three-site-per-nucleotide model for DNA with explicit ions. J. Chem. Phys. 135, 165104 22047269 10.1063/1.3652956 PMC3221706

[139] Chakraborty D., Hori N. and Thirumalai D. (2018) Sequence-dependent three interaction site model for single- and double-stranded DNA. J. Chem. Theory Comput. 14, 3763–3779 29870236 10.1021/acs.jctc.8b00091 PMC6423546

[140] Wang L., Brasnett C., Borges-Araújo L., Souza P.C.T. and Marrink S.J. (2025) Martini3-IDP: improved Martini 3 force field for disordered proteins. Nat. Commun. 16, 2874 40128232 10.1038/s41467-025-58199-2 PMC11933364

[141] Wu Y., Alessandri R., Coraor A.E., Peng X., Rico P.F.Z., Liebl K. et al. (2026) CRANBERRY: an RNA dynamics model with sugar puckering and noncanonical base pairing. bioRxiv 2026.01.12.699131, Available from: https://www.biorxiv.org/content/10.64898/2026.01.12.699131v142818444

[142] Poma A.B., Hinostroza Caldas A., Cofas-Vargas L.F., Jones M.S., Ferguson A.L. and Medrano Sandonas L. (2026) Recent advances in machine learning and coarse-grained potentials for biomolecular simulations. Biophys. J. 125, 327–343 40544328 10.1016/j.bpj.2025.06.019 PMC12968842

[143] Farré-Gil D., Arcon J.P., Laughton C.A. and Orozco M. (2024) CGeNArate: a sequence-dependent coarse-grained model of DNA for accurate atomistic MD simulations of kb-long duplexes. Nucleic. Acids. Res. 52, 6791–6801 38813824 10.1093/nar/gkae444 PMC11229373

